# Evaluation of Healthy Fit: A Community Health Worker Model to Address Hispanic Health Disparities

**DOI:** 10.5888/pcd15.170347

**Published:** 2018-04-26

**Authors:** Louis D. Brown, Denise Vasquez, Jennifer J. Salinas, Xiaohui Tang, Hector Balcázar

**Affiliations:** 1 The University of Texas Health Science Center at Houston, School of Public Health, El Paso, Texas; 2 Texas Tech University Health Sciences Center El Paso, El Paso, Texas; 3 Charles R. Drew University of Medicine and Science, Los Angeles, California

## Abstract

**Introduction:**

Hispanics in the United States have disproportionately high rates of obesity, hypertension, and diabetes and poorer access to preventive health services. Healthy Fit uses community health workers to extend public health department infrastructure and address Hispanic health disparities related to cardiovascular disease and access to preventive health services. We evaluated the effectiveness of Healthy Fit in 1) reaching Hispanic Americans facing health disparities, and 2) helping participants access preventive health services and make behavior changes to improve heart health.

**Methods:**

Community health workers recruited a sample of predominantly low-income Hispanic immigrant participants (N = 514). Following a health screening, participants received vouchers for breast, cervical, and colorectal cancer screening, and received vaccinations as needed for influenza, pneumonia, and human papillomavirus. Participants who were overweight or had high blood pressure received heart health fotonovelas and referrals to community-based exercise activities. Community health workers completed follow-up phone calls at 1, 3, and 6 months after the health screening to track participant uptake on the referrals and encourage follow-through.

**Results:**

Participants faced health disparities related to obesity and screening for breast, cervical, and colorectal cancer. Postintervention completion rates for breast, cervical, and colorectal cancer screening were 54%, 43%, and 32%, respectively, among participants who received a voucher and follow-up phone call. Among participants with follow-up data who were overweight or had high blood pressure, 70% read the fotonovela, 66% completed 1 or more heart health activities in the fotonovela, 21% attended 1 or more community-based exercise activities, and 79% took up some other exercise on their own.

**Conclusion:**

Healthy Fit is a feasible and low-cost strategy for addressing Hispanic health disparities related to cancer and cardiovascular disease.

## Introduction

Hispanics in the United States have disproportionately high rates of obesity, hypertension, and diabetes and poorer access to preventive health services (1,2). There is a need for innovative public health strategies to address the high prevalence of chronic disease and lack of access to health care among vulnerable Hispanic populations (3–5). To address these problems, federal actions like the Center for Medicaid and Medicare Services 1115 Medicaid Waivers have been launched (6). In Texas, the 1115 Waiver Program is an alternative to Medicaid expansion in association with the Patient Protection and Affordable Care Act of 2010 (7). Funding seeks to improve health care through the triple aim of improving the experience of care, enhancing population health, and reducing per capita health care costs (8). One approach to achieving the triple aim is the use of community health workers (CHWs), who can provide affordable health services in a culturally competent manner (9). Studies have demonstrated the feasibility and utility of CHWs in preventing chronic disease (10–12).

In this article, we report on a university–public health department partnership supported by Texas’s Medicaid 1115 waiver. We examined Healthy Fit, an intervention that takes a holistic approach to reducing Hispanic health disparities. Rather than targeting a single or narrow range of health problems, Healthy Fit systematically addresses disparities related to chronic disease, cancer, and access to health services. CHWs extend public health department infrastructure, providing vulnerable populations with referrals to preventive services. CHWs also provide participants with health education to understand the referrals and to help address barriers. The objectives of this study were to 1) identify the health needs of a predominantly Hispanic population living on the US–Mexico border and 2) estimate participant responsiveness to recommendations and referrals provided by CHWs that address identified health needs.

## Methods

### Study design

We used a longitudinal observational evaluation of Healthy Fit to examine a sample of 514 intervention participants from February 2015 through May 2016. The institutional review board at The University of Texas Health Science Center at Houston approved all study procedures.

### Participants

The focal population was uninsured people and Medicaid beneficiaries aged 18 years or older. We reached this population through recruitment strategies rather than by excluding participants with insurance. Our only exclusion criterion was for pregnant women, because of our interest in tracking body mass index (BMI) changes. CHWs recruited participants from community events, health fairs, and local agencies that served low-income Hispanic populations. In particular, 271 (53%) participants were recruited by CHWs at the Mexican Consulate in collaboration with their Ventanilla de Salud (health window) and 58 (11%) at Ayuda, a nonprofit community center. CHWs had a table at the Ventanilla de Salud once or twice per week and promoted the program to all consulate visitors by using the consulate’s loudspeaker announcement system. The Mexican Consulate also hosted well-attended monthly health fairs, which provided recruitment opportunities. CHWs encouraged participants to refer friends and family members via word-of-mouth, which helped to reach others of a similar socioeconomic status who might not have participated without a recommendation from someone in their social network. For the follow-up telephone interviews, CHWs called participants at different times of day and worked with their schedules to complete the interviews. CHWs made a minimum of 5 calls before conceding loss to follow-up. The response rate for follow-up on the 514 participants was 88.7%, with 456 participants reached for 1 or more telephone follow-up interviews.

### Healthy Fit intervention

The Healthy Fit intervention consisted of an initial 20- to 45-minute health screening that included referrals to clinical and community resources (eg, Take Off Pounds Sensibly [TOPS]) and 15- to 30-minute telephone follow-up interviews at 1 month, 3 months, and 6 months after the initial screening. Along with the health screening interview questions, CHWs obtained participants’ height, weight, and blood pressure. On the basis of the screening results, CHWs provided participants with referrals to each resource for which they qualified (Table 1). Each CHW conducted telephone follow-up interviews with participants to assess and encourage follow-through on the referrals provided.

**Table 1:** Referrals and Eligibility Requirements, Healthy Fit Program, El Paso, Texas, 2015–2016

Referral	Eligibility Requirements
Breast cancer screening^a^	Female aged 50–74 and No mammogram in past 2 years
Cervical cancer screening^a^	Female aged 21–64 y and No cervical cancer screening in the past 3 years and No cervical cancer screening and HPV test in past 5 years (if aged 30–64 y)
Colon cancer screening^a^	Aged 50–75 y and No FOBT in past year and No colonoscopy in past 5 years and No sigmoidoscopy in past 5 years and no FOBT in past 3 years
Pneumonia vaccine^a^	Aged ≥65 y and No pneumonia vaccine
HPV vaccine^a^	Participant aged 18–26 y or their children aged 9–26 y and No HPV vaccine
Influenza vaccine^a^	Screening date between October and March and No influenza vaccine in past year
My Heart My Community health education and exercise	BMI ≥25 or Systolic blood pressure ≥120 mm Hg or Diastolic blood pressure ≥80 mm Hg
Take Off Pounds Sensibly	BMI ≥25 and English-speaking woman aged ≥50 y
Primary care physician	Does not see a physician annually and Has health insurance

Abbreviations: BMI, body mass index; FOBT, fecal occult blood test; HPV, human papilloma virus.

a Had to be uninsured, meet federal poverty guidelines, or receive Medicaid to receive a voucher for the service.

Uninsured, underinsured, and Medicaid-eligible participants could receive vouchers for breast, cervical, and colorectal cancer screening as needed, plus influenza, pneumonia, and human papillomavirus (HPV) vaccines. We used US Preventive Services Task Force (13) guidelines to determine eligibility criteria for immunizations and cancer screenings. Participants could redeem vouchers for free services at clinics funded or reimbursed by the City of El Paso Department of Public Health. As an intermediary step in using vouchers, we tracked whether participants contacted someone about redeeming each voucher.

Participants who were overweight or had high blood pressure (according to American Heart Association guidelines [14]) received heart health fotonovelas (comic books) and physical activity resources. The fotonovelas were culturally tailored health education materials used in the Mi Corazón Mi Comunidad (My Heart My Community) curriculum, previously tested as part of Project HEART (15,16). The fotonovelas contained information and activities to improve diet and exercise, such as planning exercises during the day and dietary changes for the upcoming week; the fotonovelas required elementary literacy for comprehension. To facilitate the integration of friendship with exercise, CHWs encouraged participants to attend community-based exercises and to bring friends. CHWs shared a list of activities that were free or had a suggested donation, including Zumba classes, yoga classes, and walking groups.

### Instrument and measures

The baseline health screening instrument included questions on demographic and health status characteristics that determined eligibility for referrals. Demographic measures were age, sex, ethnicity, primary language spoken at home, employment status, yearly household income, marital status, education, country of birth, and time living in the United States. Health status measures included self-reported health, BMI (measured as weight in kilograms divided by height in meters squared), blood pressure, smoking status, and health insurance. English language proficiency was self-rated on a scale of 1 to 4, with 1 being poor and 4 being excellent. The interview followed the National Institutes of Health–funded PhenX Toolkit protocols where available (17). CHWs used a scripted follow-up telephone interview to track changes in health behaviors and use of the resources provided. At the end of each interview, CHWs encouraged participants to follow through in making health behavior changes and using the health resources provided. When pre-existing Spanish versions of the questions were not available, researchers and CHWs collaborated to translate instruments into Spanish, with back translation into English to refine the accuracy of Spanish translations (18).

### Statistical analysis

We conducted all data management and analyses in SAS version 9.4 (SAS Institute, Inc). To answer the first research question about the health needs of participants, we used the baseline health screening data. To answer the second research question on participant responsiveness to CHW referrals, we used data from the 1-, 3-, and 6-month follow-up interviews.

## Results

### Demographic characteristics

Most of the 514 participants were women (82%) and of Hispanic or Latino descent (97%); mean age was 45.9 years (range, 18–94 y) (Table 2). With respect to acculturation, 409 (80%) of participants were not born in the United States but had lived here an average 17.4 years (range, 0–85 y). Spanish was the primary language spoken at home for 89% of participants. The mean self-rated English proficiency score was 1.9 (standard deviation [SD], 1.1). Participants were predominantly low-income; 71% had a yearly household income of $19,999 or less, and more than 75% met federal poverty guidelines. Educational attainment was similarly low; 37% did not have a high school diploma and 89% did not have a bachelor’s degree.

**Table 2:** Participant Demographics and Health Characteristics (N = 514), Healthy Fit Program, El Paso, Texas, 2015–2016

Characteristic	N (%)
**Demographic**	
**Sex**	
Female	421 (81.9)
Male	93 (18.1)
Missing	0
**Hispanic or Latino descent**	
Yes	498 (96.9)
No	11 (2.1)
Missing	5 (1.0)
**Employment status**	
Employed	183 (35.6)
Unemployed	41 (8.0)
Homemaker	209 (40.7)
Other (eg, student, retired)	79 (15.4)
Missing	2 (0.4)
**Yearly household income, $**	
0–19,999	363 (70.6)
20,000–29,999	84 (16.3)
≥30,000	61 (11.9)
Missing	6 (1.2)
**Marital status**	
Married	241 (46.9)
Divorced or separated	96 (18.7)
Widowed	35 (6.8)
Never married	142 (27.6)
Missing	0
**Education**	
Less than a high school diploma	191 (37.2)
High school graduate	171 (33.3)
Some college or associate’s degree	94 (18.3)
Bachelor’s degree or higher	50 (9.7)
Missing	8 (1.6)
**Health**	
**Self-reported health **	
Poor	28 (5.5)
Fair	166 (32.3)
Good	232 (45.1)
Very good	58 (11.3)
Excellent	27 (5.3)
Missing	3 (0.6)
**Weight status (BMI^a^)**	
Underweight (<18.5)	3 (0.6)
Normal weight (18.5–24.9)	83 (16.1)
Overweight (25.0–29.9)	172 (33.5)
Obese (≥30.0)	250 (48.6)
Missing	6 (1.2)
**Blood pressure** b	
Normal	242 (47.1)
Prehypertension	172 (33.5)
Hypertension	98 (19.1)
Missing	2 (0.4)
**Smoking status**	
Never smoker	362 (70.4)
Former smoker	63 (12.3)
Smoke some days	42 (8.2)
Smoke every day	36 (7.0)
Missing	11 (2.1)
**Health insurance**	
Yes	106 (20.6)
No	407 (79.2)
Missing	1 (0.2)
**Type of health insurance**	
Private	54 (10.5)
Medicare	36 (7.0)
Medicaid	16 (3.1)
Other	8 (1.6)

a Body mass index (BMI) measured as weight in kilograms divided by height in meters squared.

b According to American Heart Association guidelines (14).

### Health status

Self-reported health was generally positive, with 317 (62%) participants reporting good to excellent health (Table 2). However, heart health risk factors such as obesity and hypertension were common. With respect to weight, 250 (49%) were obese (BMI ≥30) and 172 (34%) were overweight (BMI 25.0–29.9). For blood pressure, 172 (34%) had prehypertension and 98 (19%) had hypertension. Access to health services was limited; 407 (79%) participants were uninsured.

### Cancer screenings and immunizations

Table 3 presents data on the distribution of vouchers for preventive health services, including cancer screenings and immunizations. Approximately half of participants needed most services. For example, 83 of 158 women aged 50 to 74 years in the sample (53%) were not up to date on their breast cancer screening. The rate at which people who needed a service received a voucher ranged from 71% to 90%. Participants did not receive a voucher if they refused it, or if they did not qualify because they were not Medicaid-eligible or underinsured. In the case of breast cancer screening, 3 participants refused the breast cancer screening voucher and 5 did not qualify because of their insurance status. Thus, of the 83 women who needed a breast cancer screening, 75 (90%) received a voucher. The rates for which CHWs were able to follow up with participants to ask whether they had redeemed a particular voucher were generally high, ranging from 78% to 91% (mean, 88%) (Table 3).

**Table 3:** Use of Preventive Health Services, Cancer Screening and Vaccine Voucher Results (N = 514), Healthy Fit Program, El Paso, Texas, 2015–2016

Voucher Type	No. (% [95% Confidence Interval])				
	Needed Service/Were Age- and Sex- Eligible^a^	Received Voucher/Needed Service	Reached for Follow-Up/Received Voucher	Used Voucher/Reached for Follow-Up	Used Voucher/Needed Service
Breast cancer screening	83/158 (53 [45–60])	75/83 (90 [84–97])	68/75 (91 [84–97])	37/68 (54 [43–66])	37/83 (45 [34–55])
Cervical cancer screening	215/379 (57 [52–62])	177/215 (82 [77–87])	161/177 (91 [87–95])	69/161 (43 [35–51])	69/215 (32 [26–38])
Colorectal cancer screening	103/201 (51 [44–58])	73/103 (71 [62–80])	66/73 (90 [84–97])	21/66 (32 [21–43])	21/103 (20 [13–28])
Pneumonia vaccine	23/42 (55 [40–70])	18/23 (78 [61–95])	14/18 (78 [59–97])	2/14 (14 [0–33])	2/23 (9 [0–20])
Human papillomavirus vaccine	112/281 (40 [34–46])	93/113 (82 [75–89])	81/93 (87 [80–94])	25/81 (31 [21–41])	25/112 (22 [15–30])
Influenza vaccine	85/165 (52 [44–59])	63/85 (74 [65–83])	56/63 (89 [81–97])	19/56 (34 [22–46])	19/85 (22 [13–31])

a Numerator is the number of people who are not up to date on the particular service, based on US Preventive Services Task Force recommendations (13). Denominator is the number of people who were age- and sex-eligible for a particular service. For HPV vaccination, participants were eligible if aged 18 to 26 years or had children aged 9 to 26 years. For influenza vaccination, participants were eligible if interviewed during flu season (October 1 to March 31).

Voucher redemption rates were highest for breast cancer screening at 54%, followed by cervical cancer screening at 43%, influenza vaccination at 34%, colorectal cancer screening at 32%, HPV vaccination at 31%, and pneumonia vaccination at 14% for participants with follow-up data. Among those who needed each service, 45%, 32%, and 20% received breast, cervical, and colorectal cancer screenings, respectively. Immunizations rates were 9% for pneumonia, 22% for HPV, and 22% for influenza.

Rates of participants contacting someone about redeeming a voucher were 4 to 15 percentage points higher than rates of voucher redemption. The number and percentage of people who contacted someone about a particular service relative to the number of people receiving a voucher with follow-up data were 47 (69%) for breast cancer screening, 74 (46%) for cervical cancer screening, 24 (36%) for colon cancer screening, 3 (21%) for pneumonia vaccination, 27 (33%) for HPV vaccination, and 21 (38%) for influenza vaccination.

### Heart health

Among the 451 (88%) participants who were overweight or had high blood pressure and qualified for fotonovela and physical activity referrals, 439 (97%) received the fotonovelas and organized physical activity referrals. Twelve people who qualified were erroneously not given the referrals. Of the 439 receiving referrals, 388 (88%) have follow-up data.

Among the 388 participants with follow-up data, 273 (70%) read the fotonovela and 258 (66%) completed 1 or more of its activities. The number of fotonovela activities completed ranged from 0 to 9 (mean, 3.08; SD, 1.83) (Figure).

**Figure:**
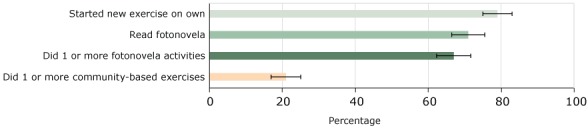
Percentage of Healthy Fit participants who completed heart health activities, among those who received heart health referrals and had follow-up data (n = 388), El Paso, Texas, 2015–2016. Error bars represent 95% confidence intervals. **Table:** 

Heart Health Activity	% (95% Confidence Interval)
Started new exercise on own	79 (75–83)
Read fotonovela	70 (66–75)
Did 1 or more fotonovela activities	66 (62–71)
Did 1 or more community-based exercises	21 (17–25)

Exercise measures among the 388 participants with follow-up data showed that 83 (21%) participated in 1 or more community-based exercise activities, completing an average of 2.87 activities per week. The most common community-based exercises were Zumba and aerobics classes. Common barriers to attending activities were lack of time, lack of interest, and activities being too far away. Another 308 (79%) took up some other exercise on their own, exercising a mean of 4.0 times per week. The most common individual exercises were walking, running, and going to the gym.

### Additional referrals

For referrals to Take Off Pounds Sensibly (TOPS), most participants were excluded because of the English-speaking requirement. Of the 11 participants referred, 1 participant (9%) attended a TOPS meeting. CHWs referred 41 participants to a primary care physician because they had health insurance but had not seen a primary care physician in the past 12 months. Of these 41 participants, 21 (51%) followed through and saw a primary care physician.

## Discussion

Healthy Fit is unique in its university–public health department partnership to address health issues disparately affecting Hispanic communities across Texas and the United States. Over 16 months, Healthy Fit reached 514 residents, offering them overdue preventive services.

### Cancer screenings and immunizations

Healthy Fit participants had substantially lower rates of cancer screening at baseline compared with the national average, suggesting the CHWs were effective in recruiting people facing disparities in access to cancer screenings. Before the intervention, 47% of participating women aged 50 to 74 had received a breast cancer screening in the past 2 years, compared with a national rate of 67% (19). Similarly, 43% of participating women met the screening guidelines for cervical cancer screening or Papanicolaou compared with 69% nationally (19). Finally, 49% of eligible participants had any of the 3 tests to detect colon cancer (sigmoidoscopy, colonoscopy, fecal occult blood test), which is also lower than the US average of 59% (19).

Healthy Fit was successful in addressing these cancer-screening disparities. Postintervention cancer-screening uptake among participants receiving a service voucher and follow-up was 54% for breast, 43% for cervical, and 32% for colorectal cancer. Uptake rates in previous studies of cancer screening programs vary but are similar to our findings. For example, Larkey et al reported uptake rates of 34% for breast, 45% for cervical, and 9% for colorectal cancer screening (20). Participants often mentioned lack of time, lost vouchers, and transportation difficulties as barriers to cancer screening. More postreferral support from the CHWs could improve participant follow-through on obtaining screenings, such as help in setting appointments and navigating transportation problems.

Unlike cancer screening, Healthy Fit participants’ HPV, pneumonia, and influenza immunization rates were mixed compared with national rates. Participants had immunization rates of 45% for pneumonia, 60% for HPV, and 48% for influenza, compared with national rates of 64% for pneumonia vaccination, 42% for HPV (among women aged 19–26 y), and 45% for influenza (2).

Postintervention uptake of the immunizations was lower than for cancer screenings, at 14% for pneumonia, 31% for HPV, and 34% for influenza. The most commonly reported reasons for not using the vaccine vouchers were lack of time and indecision. More in-depth conversations that work to resolve the ambivalence about getting a vaccination may help to improve participant follow-through.

Although Healthy Fit was not able to address all screening and immunization barriers, it addressed key economic and insurance coverage barriers, substantially improving the reach of preventive services. Additionally, CHWs helped assuage personal barriers, such as embarrassment and fear of cancer screening. CHWs worked to find culturally sensitive solutions, such as asking a family member for a ride to the clinic. CHW strengths included reading facial expressions to adjust information delivery. These CHW capacities provide key advantages over other prevention strategies, such as computerized clinical decision support systems (21). Overall, the low-cost strategy used by Healthy Fit demonstrates efficacy in reaching a vulnerable population to increase their cancer screening and immunization rates.

### Heart health

CHWs reached residents with a disproportionally high rate of obesity of 49%, compared with 35% for US adults overall and 42% for Hispanic adults (22). Nearly 9 of 10 participants received referrals to improve diet and exercise because they were overweight or had high blood pressure. Results suggest the fotonovelas were well received, with most participants reading and completing one or more fotonovela activities. Some of the most common actions reported by participants were decreased salt consumption and taking blood pressure medications as instructed. This simple and low-cost strategy for increasing heart health education among a Hispanic population with low educational attainment is encouraging.

CHWs also worked with participants to identify nearby community-based physical activities that were free or had a suggested donation; 21% of participants attended. Although lower than fotonovela uptake rates, 21% is a substantial portion of participants and may represent a sustainable behavior change if the social nature of the activities promotes a sense of belonging (23). The adoption of exercise routines outside of the community-based activities was high, with nearly 4 of 5 participants taking on new exercises. The increased exercise is critical, given its tremendous health benefits, which address participants’ health disparities (24). The relatability of the CHWs and their straightforward advice may have helped to facilitate participant follow-through on the heart health referrals.

### Limitations and future directions

One limitation of this study is that the success of our approach may vary by community or ethnicity. We reached a predominantly low-income Hispanic immigrant population living on the US–Mexico border. Future research can examine the generalizability of our approach. Another limitation is our reliance on self-reported outcomes. For example, participants may have overreported exercise and other health behaviors targeted by the intervention. To be more objective, future research can collect service provider data on screenings and immunizations, along with attendance data from the community-based exercise activities. A third limitation is that most participants were women, which is often the case in CHW interventions (25). Reaching men, particularly Hispanic men, is a challenge to address; using male CHWs could help (26). A fourth limitation is that the rates of those who used the voucher and needed a particular service provide a conservative estimate of program ability to help people acquire needed services. These rates may underestimate participant receptiveness to the CHW referrals, as we did not follow up with participants about a service if they did not receive a voucher. Further, the rate assumes those without telephone follow-up data did not receive a service, even though service use is unknown.

### Conclusion

Healthy Fit was successful in reaching and following up with a vulnerable Hispanic population that faced substantial health disparities. The intervention addressed identified disparities, helping a substantial percentage of participants obtain needed cancer screenings and immunizations, participate in heart health activities, and adopt new exercise routines. The results provide promise for 1115 Medicaid waiver programs as an effective approach to chronic disease prevention among the uninsured. The preventive services delivered may avert more costly health problems (27). In sum, findings suggest Healthy Fit is feasible and could be implemented on a larger scale, providing an economically viable approach to reducing Hispanic health disparities.
